# Influence of Refractive Condition on Retinal Vasculature Complexity in Younger Subjects

**DOI:** 10.1155/2014/783525

**Published:** 2014-10-13

**Authors:** Mohd Zulfaezal Che Azemin, Norsyazwani Mohamad Daud, Fadilah Ab Hamid, Ilyanoon Zahari, Abdul Halim Sapuan

**Affiliations:** ^1^Department of Optometry and Visual Science, Kulliyyah of Allied Health Sciences (KAHS), International Islamic University Malaysia (IIUM), Bandar Indera Mahkota, 25200 Kuantan, Pahang, Malaysia; ^2^Department of Diagnostic Imaging and Radiotherapy, Kulliyyah of Allied Health Sciences (KAHS), International Islamic University Malaysia (IIUM), Bandar Indera Mahkota, 25200 Kuantan, Pahang, Malaysia

## Abstract

*Objective*. The aim of this study was to compare the retinal vasculature complexity between emmetropia, and myopia in younger subjects. *Methods*. A total of 82 patients (24.12 ± 1.25 years) with two types of refractive conditions, myopia and emmetropia were enrolled in this study. Refraction data were converted to spherical equivalent refraction. These retinal images (right eyes) were obtained from NAVIS Lite Image Filing System and the vasculature complexity was measured by fractal dimension (*D*_*f*_), quantified using a computer software following a standardized protocol. *Results*. There was a significant difference (*P* < 0.05) in the value of *D*_*f*_ between emmetropic (1.5666 ± 0.0160) and myopic (1.5588 ± 0.0142) groups. A positive correlation (rho = 0.260, *P* < 0.05) between the *D*_*f*_ and the spherical equivalent refraction was detected in this study. Using a linear model, it was estimated that 6.7% of the variation in *D*_*f*_ could be explained by spherical equivalent refraction. *Conclusions*. This study provides valuable findings about the effect of moderate to high myopia on the fractal dimension of the retinal vasculature network. These results show that myopic refraction in younger subjects was associated with a decrease in *D*_*f*_, suggesting a loss of retinal vessel density with moderate to high myopia.

## 1. Introduction

Human retina can be easily visualized and observed using a fundus camera that produces a high quality image. The use of this special camera enables the use of retinal image as a means for diagnosing vascular-related conditions. It is therefore not surprising that the retina has been extensively explored by researchers in various areas [1–3]. The retinal image has been widely used and has great significance in detecting various eye diseases which include the diabetic retinopathy [4], hypertensive retinopathy [5], and glaucoma [6]. It is also shown that the retinal vascular changes reflect the microvascular pathologies such as systemic hypertension and stroke [2, 7].

Retinal vasculature complexity is a complex branching pattern of vessels which cannot be described using Euclidean geometric pattern. Fractal represents a type of non-Euclidean shape which has been widely used in many aspects of medicine to describe statistically similar branching biological structures. The application of fractal analysis was pioneered by Mandelbrot [8]. The concept of fractal analysis is summarized in the following quotation from Mandelbrot: “Clouds are not spheres, mountains are not cones, coastlines are not circles, and bark is not smooth, nor does lightning travel in a straight line.” The fractal analysis has been widely been used to describe objects from trees to coastlines to vascular system in human body which includes the retinal microcirculation [9]. Fractal curves can be quantified using fractal dimension, a noninteger unit between 1 and 2, with higher integer reflecting the increased of complexity as represented by the density of the space-filling pattern of the retinal vascular tree.

The greater the value of the fractal dimension, the higher the complexity of the structure which indicates proliferation in the microvascular system, whereas the lower the fractal dimension, the less complex and less dense the vessels branching network [10, 11].

An important methodological consideration in quantitative measurement of retinal vasculature complexity is the effect of refractive error, lens opacity, and aging [3, 12]. It is important to understand the influence of these parameters on the measurement of retinal fractal dimension. Most studies in fractal dimension have only been performed in the older population [12–14]. However, aging is one of the most common risk factors for multiple conditions and it may dilute the effect of the condition under investigation [15, 16]. To address this issue, this study will focus on the comparison of the retinal vasculature complexity measurements in younger subjects.

## 2. Materials and Methods

The initial retinal image samples consisted of 852 right eye retinal images collected from those who attended IIUM Optometry Clinic at various time points from January 2009 till June 2012 as reported in the previous study [3]. Informed consent was obtained from the subjects and approval from the local ethics committee was granted in accordance with the principles laid down by the Declaration of Helsinki. The patient's medical records containing their respective retinal images were explored to extract the patient's data. A total of 100 retinal images were chosen and further classified into two groups which are moderate to high myopia and emmetropia groups based on the spherical equivalent refraction (SER) and other inclusion criteria. SER was defined as the sum of a spherical power plus half cylindrical power refraction. For this study, the baseline refractive power was obtained based on the latest subjective refraction in the participant's record. The baseline refraction was then converted into SER. Emmetropic group was defined as SER between +1.00 DS and −1.00 DS. Moderate to high myopic group was defined as the spherical equivalent refraction from −3.00 DS and less.

Age was defined at the time the retinal photograph was captured. The samples were conveniently chosen by considering the image quality. Out of the 100 samples, 18 (18%) samples were excluded due to inconsistent cropping region with others or deemed ungradable by the grader. Inclusion criteria were considered to ensure that the results will not be influenced by other confounders. The inclusion criteria were as follows.

1. Age 18–30 years old: this range of age was selected in order to avoid the effect of the aging process [3].
2. Emmetropia (SER between −1.00 D and +1.00 D) or moderate to high myopia (SER −3.00 D or worse) [12].
3. No lens opacity. Fractal dimension is significantly affected by lens opacity [12].
4. No significant underlying systemic disorder. Diabetic retinopathy and hypertensive retinopathy may affect the retinal vasculature network [17].
5. No history of ocular trauma.

Table 1 summarizes the characteristics of subjects who were included or excluded from the analysis. There were no significant differences in the characteristics of the subjects between the two groups.

Readily available fundus images were obtained from NAVIS-Lite Filing System. The images comprised of 45-degree field of view captured by non-mydriatic Auto Fundus Camera AFC-230/210. The region of interest is centered midway between macula and optic disc. The region of interest will be assumed to be consistent when employing 45-degree field of view for all retinal images.

Right eye retina images were used in this study, as a previous study reported that there is a similar linear decline of fractal dimension with increasing age regardless of whether the data are from the right or left eye [11]. This 45-degree field of view uses approximately the midway of the macula and optic disc as a centre of the fundus image.

The vessels were segmented using custom-written software to remove nonvessels from the image. We used the methodology developed earlier [3] for this purpose. Briefly, the algorithm was trained to recognize pixels associated with blood vessels (white pixels) and nonblood vessels (black pixels) using a supervised vessel segmentation technique. The remaining artefacts were then deleted by a trained grader to ensure that only vessels were being analyzed.

**Figure 1:**
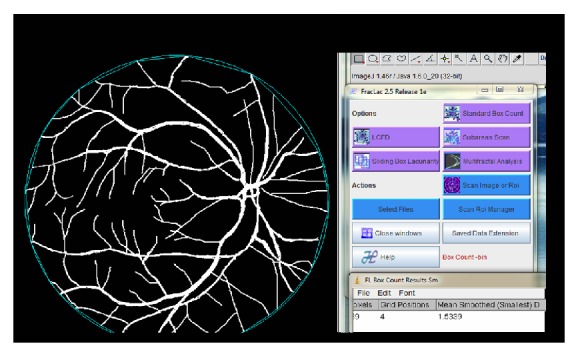
Measurement of fractal dimension (*D*_*f*_) using ImageJ.

The term “retinal vasculature complexity” used in this study refers to the value of the fractal dimension (*D*_*F*_). *D*_*F*_ was calculated using publicly available software, Fraclac, a plugin for the ImageJ program [18]. The general procedure for the box-counting technique is to systematically lay a series of grids of decreasing size, *S*, over the vasculature image and calculate the number of grids, *N*, that include the vasculature (white pixels). The fractal dimension can be calculated from the slope of log *N* versus log *S*.  Figure 1 illustrates the screenshot of the software.

## 3. Statistical Analysis

Statistical Package for the Social Science (SPSS) was employed for the statistical analyses. Shapiro-Wilk test was applied to test the normality of the samples. Independent sample *t*-test was used to compare the *D*_*f*_ between emmetropic and myopic groups. Correlation analysis was also performed using Pearson correlation to see the trend between *D*_*f*_ and SER followed by simple linear regression to investigate the relationship between fractal dimension changes in response to the SER. Continuous data were used to run correlation and simple linear regression analysis. A value of *P* < 0.05 was considered significant.

## 4. Result

The final 82 gradable samples selected for analysis were distributed as shown in Table 2. The overall *D*_*f*_ mean for the 82 samples was calculated to be 1.5627 with a standard deviation of 0.015.  Table 3 shows mean *D*_*f*_ for the emmetropic and myopic groups. There was a significant difference (*P* < 0.05) in the value of fractal dimension for emmetropic group (*D*_*f*_ = 1.5666 ± 0.0160) and myopic group (*D*_*f*_ = 1.5588 ± 0.0142). These results suggest that fractal dimension of myopia tends to have smaller *D*_*f*_ compared to the emmetropia group.

Correlation analysis shows that there is a significant correlation between spherical equivalent refraction and fractal dimension (*r* = 0.260, *P* < 0.05). There is a fair positive correlation of *D*_*f*_ with SER.

The result of the simple linear regression suggests that a significant proportion of the total variation in fractal dimension was predicted by spherical equivalent refraction (*P* = 0.018). It is observed that, for every 1 unit increase in spherical equivalent refraction, fractal dimension will increase to 0.002 units. *R*-squared analysis indicates that approximately 6.7% of the variation in fractal dimension was predicted by SER. The SER itself only explained a small proportion of the variance of the fractal dimension. Other factors, for example, physical activity and dietary intake, may influence the rarefaction of the microvasculature, as demonstrated in the previous works [19, 20].

## 5. Discussion

The study retinal vasculature complexity and its related issues have not been widely explored and studied. To our knowledge, this is the first study to find relationship between retinal vasculature complexity and refractive conditions (myopia and emmetropia) in younger population. This section will discuss in details the study findings and the use of spherical equivalent as potential confounder to the fractal dimension measurement. There are a number of studies on fractal dimension published in the past few years. However, the study on relationship between fractal dimension and refractive error is very limited.

The degree of retinal vasculature complexity can be quantified by a single number which is fractal dimension. In this study, the mean value of fractal dimension of the overall population is 1.5627 ± 0.0155. Previous studies have reported the mean for fractal dimension of human retinal vessel to be 1.444 ± 0.023 [12], 1.437 ± 0.025 [21], 1.408 ± 0.025 [22], and 1.7 [9].

The inconsistency of the results probably occurred due to different methods used by different researchers. The variations found in the literature in the fractal dimension values maybe due to the use of retinal image with different resolution, different location of the cropping region, methods used in image acquisition (e.g., red-free, colour, or fluorescein images), methods of segmentation, and methods to calculate the fractal dimension.

Another possible explanation for the differences in the value of fractal dimension may be due to the demographic characteristics. In one similar study [12], their participants were those from suburban Australian population aged 49 years and above while this study focused on the *D*_*f*_ analysis on younger group of subjects.

Myopic group tends to have smaller *D*_*f*_ compared to the emmetropic group. This finding is in agreement with Li et al. [12] which showed that eyes with high myopia tended to have smaller mean *D*_*f*_. However, the exact mechanism involved for promoting this effect is still uncertain.

It is interesting to note that there is a significant correlation between spherical equivalent refraction and fractal dimension (*P* < 0.05). The fractal dimension (*D*_*f*_) showed a fair positive correlation (rho = 0.260) with spherical equivalent refraction and approximately 6.7% of the variation in fractal dimension is associated with refractive error. These results are supported by the findings of Li et al. [12] which accounted that less than 3.5% of the variation in fractal dimension is associated with refractive error and this signifies the minimal changes of refractive error on fractal dimension value. The minimal slope of 0.0016/D was presented in their study and showed the linear association between spherical equivalent power and mean *D*_*f*_ in eyes with hyperopia, emmetropia or myopia. Rarefaction of retinal vasculature is presented in high myopia which can be seen from the apparent reduction of mean *D*_*f*_ with myopia greater than 4D.

The current study demonstrates the reduction of fractal dimension towards more myopic refraction. As stated by Li et al. [12], the thinning of retinal tissue in myopia due to excessive elongation of the eye affected the apparent vessel's diameter may cause smaller vessels to be undetected by the blood vessel segmentation software. Besides that, Shimada et al. [23] also reported that excessive elongation of the eyeball in high myopia causes mechanical expansion and thinning of the retina, which might result in the straightening and decreased diameter of the retinal vessels (narrowing of blood vessels). The thinning and atrophy of the retinal tissues might decrease the need for oxygen and consequently decrease blood circulation.

One limitation of this study is the use of retrospective data which caused the absence of the other potential confounders which include anthropometric data, blood pressure measurement, glucose level, physical activity, and dietary intake that may affect the result. However, we have minimized the problem with the inclusion criteria introduced at the outset of the study.

## 6. Conclusion

This study provides valuable findings about the association of moderate to high myopia and the rarefaction of the retinal vasculature network in younger subjects which confirms the findings from the previous study in older population. While the mechanism of the decreased of retinal vessel density in highly myopic eyes is uncertain, similar mechanisms might underlie the decreased retinal blood flow in highly myopic eyes with thinning of the retina [23]. These results show that myopic refraction in younger subjects was associated with a decrease in *D*_*f*_, suggesting a loss of retinal vessel density with moderate to high myopia. To further validate this finding in subjects from different demographics, a multicentre study needs to be performed.

## Figures and Tables

**Table 1:** Summary of the characteristics of subjects included and excluded from the fractal dimension analysis.

Characteristics	Included subjects (*n* = 82)	Excluded subjects (*n* = 18)	*P* value
Gender (male)	42.6%	50.0%	0.571
Age (years)	24.03 ± 2.30	24.33 ± 2.47	0.625
Spherical equivalent refraction (diopter)	−2.46 ± 2.68	−2.52 ± 2.82	0.923

**Table 2:** Distribution of the subjects according to age and spherical equivalent refraction (SER).

Group	Number of subjects	Age mean ± SD (years)	SER mean ± SD (diopter)
Emmetropes	41	24.27 ± 2.49	−0.10 ± 0.50
Myopes	41	23.93 ± 2.13	−4.70 ± 1.64

**Table 3:** Comparison of *D*_*f*_ between emmetropic and myopic groups.

	Emmetropia mean (SD)	Myopia mean (SD)	Mean difference (95% CI)	*P* value
Fractal dimension (*D*_*f*_)	1.5666 (0.0160)	1.5588 (0.0142)	0.0079 (0.0012, 0.0145)	0.021
